# Radiation Dose Reduction during Radial Cardiac Catheterization: Evaluation of a Dedicated Radial Angiography Absorption Shielding Drape

**DOI:** 10.5402/2012/769167

**Published:** 2012-09-04

**Authors:** Andrew Ertel, Jeffrey Nadelson, Adhir R. Shroff, Ranya Sweis, Dean Ferrera, Mladen I. Vidovich

**Affiliations:** ^1^Division of Cardiology, University of Illinois at Chicago Medical Center, Chicago, IL 60612, USA; ^2^Department of Medicine, Roger Williams Medical Center, Boston University School of Medicine, Providence, RI 02908, USA; ^3^Department of Medicine and the Bluhm Cardiovascular Institute, Northwestern University Feinberg School of Medicine, Chicago, IL 60611, USA

## Abstract

*Objectives*. Radiation scatter protection shield drapes have been designed with the goal of decreasing radiation dose to the operators during transfemoral catheterization. We sought to investigate the impact on operator radiation exposure of various shielding drapes specifically designed for the radial approach. *Background*. Radial access for cardiac catheterization has increased due to improved patient comfort and decreased bleeding complications. There are concerns for increased radiation exposure to patients and operators. *Methods*. Radiation doses to a simulated operator were measured with a RadCal Dosimeter in the cardiac catheterization laboratory. The mock patient was a 97.5 kg fission product phantom. Three lead-free drape designs were studied. The drapes were placed just proximal to the right wrist and extended medially to phantom's trunk. Simulated diagnostic coronary angiography included 6 minutes of fluoroscopy time and 32 seconds of cineangiography time at 4 standard angulated views (8 s each), both 15 frames/s. ANOVA with Bonferroni correction was used for statistical analysis. *Results*. All drape designs led to substantial reductions in operator radiation exposure compared to control (*P* < 0.0001). The greatest decrease in radiation exposure (72%) was with the L-shaped design. *Conclusions*. Dedicated radial shielding drapes decrease radiation exposure to the operator by up to 72% during simulated cardiac catheterization.

## 1. Introduction

 Radial artery access for cardiac catheterization offers an attractive alternative to transfemoral access for coronary angiography and percutaneous coronary intervention as it has been associated with fewer access site complications, a reduction in major bleeding events and shorter hospital duration [1–4]. However, radial access is associated with increased radiation exposure to the operator due to longer fluoroscopy times and closer positioning of the operator to the X-ray source [5–9]. Cumulative, low-dose radiation exposure is associated with adverse effects on skin and a small increased risk of certain types of cancer [10, 11]. The increased radiation dose associated with radial access has raised concerns among interventional cardiologists and limited the widespread adoption of this approach for diagnostic and interventional cardiac catheterization procedures, despite the advantages radial access offers to the patient. While standard radiation protection equipment and shielding methods offer a substantial reduction in scatter radiation exposure to interventional cardiologists, risk remains. There has been limited investigation into optimal radiation shielding in the cardiac catheterization laboratory, particularly with regard to the transradial approach. The aim of the current study is to evaluate the additional reduction in scatter radiation to the operator provided by three novel lead-free drapes designed specifically for transradial cardiac catheterization when used in addition to standard shielding methods.

## 2. Materials and Methods

**Figure 1:**
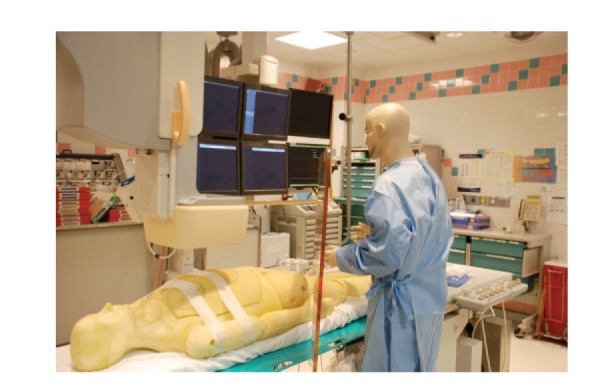
Simulated laboratory setup.

The study was performed using a Philips Allura Xper FD10/10 Fluoroscopy device (Philips Healthcare, Andover, Massachusetts) equipped with an MRC-G5 0508 Maximus Rotalix ceramic X-ray tube (Philips Medical, Hamburg, Germany). The setup for the simulated cardiac catheterizations is shown in Figure 1 and described below. The mock patient is a 97.5 kg fission product phantom (phantom ID # PL-201, provided by the Department of Energy, Idaho Falls, ID) composed primarily of the tissue-substitute adiprene. It was placed on the catheterization table in the supine position. The phantom consists of a full anthropomorphic body with full skeletal structures including articulated limbs, a full complement of simulated internal organs, and a simulated head and neck [12]. 

A mock operator composed of a 180 cm-tall mannequin was positioned at a distance from the table consistent with the primary operator position for a right radial cardiac catheterization. The mock operator was equipped with a standard lead apron and thyroid guard (0.5 mm Pb equivalent). In addition, conventional radiation shielding materials including a moveable lead shield (Mavig 0.5 mm Pb shield, Model OT25B05, Munich, Germany) positioned just proximal to the radial insertion site at a 45 degree angle with respect to the table and a lead skirt (0.5 mm Pb equivalent) extending downward to the floor from the base of the table were utilized in each simulated cardiac catheterization. 

All imaging was acquired with fluoroscopy set to “low” (15 frames/sec, 4 rad/min, max 120 kV), cinematic acquisition imaging frame rates at 15 frames/sec, source to image distance at 100 centimeters, focal distance at 20 cm, and table height at 85 cm. Images were acquired in the standard manner with the assistance of an experienced radiographer behind a transparent 1.0-cm lead screening between operator and mock patient and adhering to the American Association of Physicists in Medicine's guidelines for proper use of ionizing radiation equipment in the cardiac catheterization laboratory [13]. Each trial run consisted of three fluoroscopy views at 2 minutes each (center of the chest, left anterior oblique (LAO) 30 degrees and right anterior oblique (RAO) 30 degrees) and four cineangiography views at 8 seconds per angulated view (LAO 30 Caudal 30, AP Caudal 30, LAO 30 Cranial 30, and RAO 30 Cranial 30). Two experimental runs were completed for a control and each of the 3 lead-free drapes.

**Figure 2:**
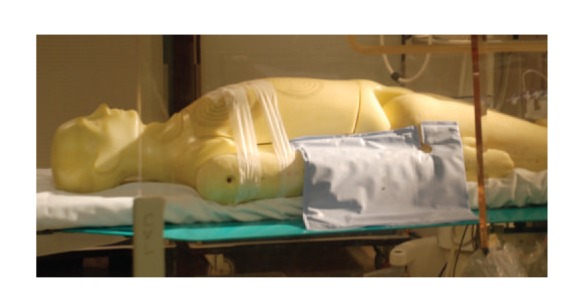
Drape 1.

**Figure 3:**
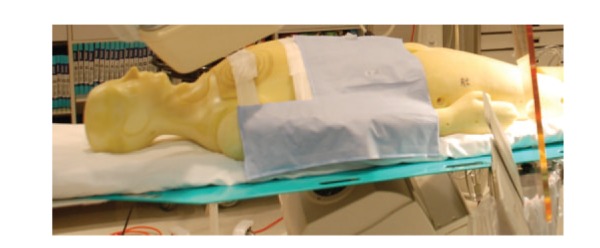
Drape 2.

**Figure 4:**
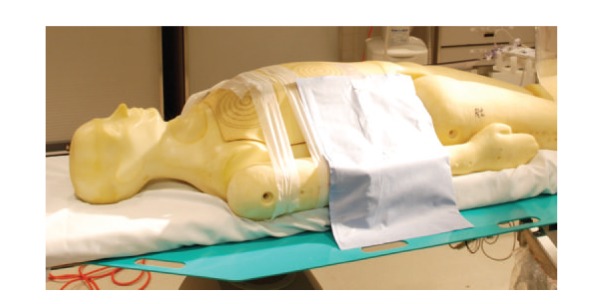
Drape 3.

The lead-free drapes that were placed on the phantom were custom-made for cardiac catheterizations that utilized right radial arterial access. Three different drapes were used in the study (Figures 2, 3, and 4): drape SX7230 measures  13′′ × 16′′  (Drape 1), drape ADS0971 is an L-shaped drape measuring 16′′ × 20′′ (Drape 2) and drape SX7200 measures 12′′ × 16.5′′ (Drape 3) (AngioSystems, Inc., Ducktown, TN, USA). The drapes were composed of a proprietary blend of metals and minerals including tungsten, tin, antimony, cerium oxide and trace metal other than lead and encased in a phthalate-free flexible binding material. The drapes provided 91% attenuation at 90 kVP with 0.28 mm lead-equivalency thickness (data on file, AngioSystems, Inc.). During the trial runs, the drapes were placed just proximal to the right wrist and extended medially to the phantom's trunk (Figures 2–4).

Ionizing radiation was measured with a RadCal Dosimeter (model 9010, RadCal Corp., CA, USA) and a Leakage/Low Level Measurements Chamber (model 10x5-180, cross-section 100 cm, volume 180 cm^3^) placed in the center of the mock operator's chest over the lead apron. After each experimental run, cumulative radiation in mGy was recorded from the dosimeter. In addition, to ensure equal radiation exposure between various measurements, recordings were made of the dose area product (DAP) (Gycm^2^ and air kerma (mGy).

Statistical analysis was performed using SPSS (SPSS, Inc., Version 19.0, Chicago, IL, USA). ANOVA with Bonferroni correction was used. A *P* value of <0.05 was considered statistically significant.

## 3. Results

**Figure 5:**
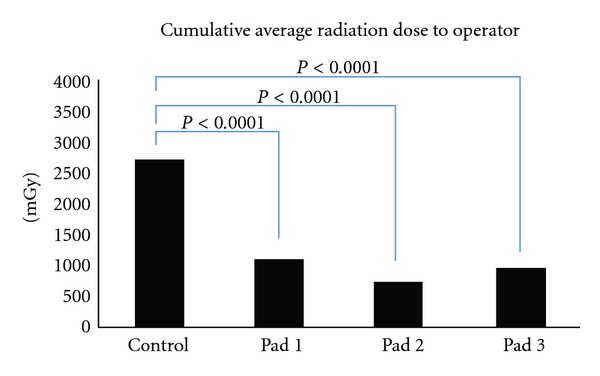
Cumulative average radiation dose to operator.

A total of eight simulated diagnostic right radial cardiac catheterizations were carried out, comprised of two control runs and two trials with each of the three radial radiation drapes. Despite identical fluoroscopy and cineangiography times, radiation exposure to the operator was significantly reduced with each radiation drape as compared to the control, with the greatest reduction being seen with the L-type drape (Drape 2) (Figure 5). The relative reduction in radiation exposure was 58% with Drape 1 (2681 ± 41 mGy versus 1122 ± 79 mGy, *P* < 0.0001), 72% with Drape 2 (2681 ± 41 mGy versus 747 ± 31 mGy, *P* < 0.0001), and 65% with Drape 3 (2681 ± 41 mGy versus 927 ± 105 mGy, *P* < 0.0001). Average cumulative radiation dose for the control and each drape is shown in Table 1. To assess for differences in radiation dose delivered by the X-ray generator, dose area product (DAP) was measured for each of the eight simulated diagnostic catheterizations, and no significant differences were found (DAP = 34,468 ± 2153 Gy cm^2^, *P* = 0.395).

Significant differences were found in radiation exposure to the operator as a result of different camera angulations during cineangiography. For the control runs, the LAO 30 cranial 30 projection delivered a significantly higher radiation dose than any other angulation, resulting in approximately eight times the dose delivered by the next highest angulation, RAO 30 cranial 30 (1432 ± 54 mGy versus 177 ±  1 mGy, *P* < 0.0001). The reduction in radiation exposure to the operator provided by the radiation drapes was greatest in the LAO 30 cranial 30 position, and Drape 2 consistently displayed the highest level of radiation reduction. In this angulated projection, the relative reduction in radiation exposure to the operator achieved by Drape 2 was 86% (1432 ± 54 mGy versus 205 ± 14 mGy, *P* < 0.0001).

A total of 6 minutes of fluoroscopy time and 32 seconds of cineangiography time were used in the simulated cardiac catheterizations. Radiation exposure to the operator was significantly greater from cineangiography as compared to fluoroscopy during the control simulations (1915 ± 57 mGy versus 766 ± 16 mGy, *P* < 0.0001). With the use of Drape 2, the difference in radiation exposure between cineangiography and fluoroscopy was reduced (466 ± 22 mGy versus 281 ± 8.3 mGy, *P* = 0.115). 

## 4. Discussion

In the United States, a majority of cardiac catheterizations are carried out with arterial access achieved via the femoral artery; however, the number of catheterizations utilizing transradial access is increasing as this approach has been shown to reduce complication rates and reduce hospital stay [1–4]. Despite these advancements, adoption of transradial catheterization has been hampered at least in part by a growing body of evidence demonstrating an association of transradial catheterization with a significant increase in radiation exposure to both patients and operators [5–9]. This increase in radiation can be largely accounted for by the increase in fluoroscopy time due to the technical challenges posed by transradial access, particularly in inexperienced operators [14]. In addition, standard radiation protection devices utilized in the cardiac catheterization laboratory were designed for transfemoral access and are not optimized for the transradial approach.

Although underestimated by interventional cardiologists for a long time, the risks posed by radiation exposure in the cardiac catheterization laboratory are a growing area of concern [9]. Chronic, low-dose radiation exposure such as that present in the cardiac catheterization laboratory has been associated epidemiologically with a small but nonnegligible increased risk of certain types of cancers [10]. While no conclusive evidence to date has linked radiation exposure in the cardiac catheterization laboratory to an increased risk of cancer, risk prediction models estimate that the lifetime attributable risk of cancer for the most exposed staff in the cardiac catheterization laboratory is increased [10].

 In the last twenty years, radiation doses to primary operators in cardiac catheterization laboratories have not changed [15]. While improvements have been made in recent years in reducing the scatter radiation emitted by fluoroscopy/cineangiography equipment, the expected reduction in radiation dose to the operator is likely being offset by the increased complexity of cases that are undertaken in contemporary cardiac catheterization laboratories. This inability to impact radiation exposure to the operator highlights the need for alternative shielding techniques to reduce radiation exposure to operators.

It has previously been shown that radiation scatter reduction drapes significantly reduce radiation exposure to patients and operators during interventional fluoroscopic procedures [16]. In a recent trial investigating optimized conventional shielding in a simulated cardiac catheterization environment utilizing right femoral access, results suggest that up to an 80% reduction in scatter radiation is achievable with optimal use of radiation shielding. In that trial, conventional shielding included a moveable upper body lead shield, lower body lead skirt with vertical extension as well as a scatter reduction drape placed in the conventional position for femoral access [17]. In a recent randomized trial in humans, a lead-free scatter reduction drape was shown to reduce radiation exposure to the operator by 23% during transradial cardiac catheterization when used in addition to conventional shielding [18]. In both of these trials, the scatter reduction drapes were designed for transfemoral access and not specifically modified for the radial approach. 

The findings of the current study underscore the marked impact that optimized radiation shielding can have on the exposure of interventional cardiologists to potentially hazardous ionizing scatter radiation. Our study is the first to specifically evaluate the radiation scatter reduction achievable by a lead-free shielding drape *specifically* designed for a right radial cardiac catheterization, and demonstrates that a unique L-type radial radiation drape can reduce radiation exposure to the operator by up to 72% in a simulated diagnostic cardiac catheterization. In addition, our study reveals several modifiable factors that had a significant impact on radiation exposure to the operator. Changes in camera angulation are known to impact radiation exposure. Interestingly, we found that the LAO 30 cranial 30 projection was responsible for over 50% of the scatter radiation exposure to the operator during control runs. We also measured the differences in radiation exposure produced by fluoroscopy and cineangiography. Cineangiography, although accounting for less than 15% of the total radiation exposure time during simulated cardiac catheterization, accounted for >70% of the radiation exposure to the operator during control runs. Knowledge of these types of variables, in addition to optimized radiation shielding, provides cardiac catheterization operators the tools to minimize scatter radiation exposure and better protect themselves, the patient, and the lab staff. 

The current study is limited by several factors. The study was performed in a simulated cardiac catheterization lab environment and as such cannot fully account for the variables and conditions present in a working clinical laboratory. In addition, the dosimeter was not placed underneath a standard lead apron on the mock operator, the result of which may be an overestimation of the radiation scatter dose reduction provided by the lead-free drapes with regard to the operator's torso and thyroid. Further randomized trials are needed to evaluate the efficacy of optimal radiation protection for the operator during cardiac catheterization performed through a transradial approach.

In summary, our results address an important gap in the literature showing that in a simulated cardiac catheterization lab setting, while controlling for fluoroscopy and cineangiography time, radiation drapes designed specifically for transradial cardiac catheterization significantly reduce operator radiation exposure when used in addition to standard radiation protection. 

## Figures and Tables

**Table 1:** Average cumulative radiation dose.

	Control (mGy)	Drape 1 (mGy)	Drape 2 (mGy)	Drape 3 (mGy)
Center of chest	371.35	171.6	118.25	211.05
LAO 30 CRAN 0	650.8	247.2	212.25	277.7
RAO 30 CRAN 0	766.2	347.65	280.6	357.65
LAO 30 CAUD 30	914.95	456.6	358.9	459.45
RAO 0 CAUD 30	1072	546.45	420.75	525.4
LAO 30 CRAN 30	2504	958.25	625.8	795.75
RAO 30 CRAN 30	2681	1122	746.8	927.9

Average dose reduction	** **	58.14%	71.77%	65.39%

LAO: Left anterior oblique.

RAO: Right anterior oblique.

CAUD: Caudal.

CRAN: Cranial.

## Abbreviations

LAO: Left anterior oblique
RAO: Right anterior oblique
mGy: Milligray
DAP: Dose area product.
